# Progress Toward Hepatitis B and Hepatitis C Elimination Using a Catalytic Funding Model — Tashkent, Uzbekistan, December 6, 2019–March 15, 2020

**DOI:** 10.15585/mmwr.mm6934a3

**Published:** 2020-08-28

**Authors:** Rick Dunn, Erkin Musabaev, Homie Razavi, Shakhlo Sadirova, Shokhista Bakieva, Katie Razavi-Shearer, Krestina Brigida, Saleem Kamili, Francisco Averhoff, Muazzam Nasrullah

**Affiliations:** ^1^Center for Disease Analysis Foundation, Lafayette, Colorado; ^2^Research Institute of Virology, Tashkent, Uzbekistan; ^3^Division of Viral Hepatitis, National Center for HIV/AIDS, Viral Hepatitis, STD and TB Prevention, CDC.

In 2016, the World Health Organization (WHO) set hepatitis elimination targets of 90% reduction in incidence and 65% reduction in mortality worldwide by 2030 (*1*). Hepatitis B virus (HBV) and hepatitis C virus (HCV) infection prevalences are high in Uzbekistan, which lacks funding for meeting WHO’s targets. In the absence of large financial donor programs for eliminating HBV and HCV infections, insufficient funding is an important barrier to achieving those targets in Uzbekistan and other low- and middle-income countries. A pilot program using a catalytic funding model, including simplified test-and-treat strategies, was launched in Tashkent, Uzbekistan, in December 2019. Catalytic funding is a mechanism by which the total cost of a program is paid for by multiple funding sources but is begun with upfront capital that is considerably less than the total program cost. Ongoing costs, including those for testing and treatment, are covered by payments from 80% of the enrolled patients, who purchase medications at a small premium that subsidizes the 20% who cannot afford treatment and therefore receive free medication. The 1-year pilot program set a target of testing 250,000 adults for HBV and HCV infection and treating all patients who have active infection, including those who had a positive test result for hepatitis B surface antigen (HBsAg) and those who had a positive test result for HCV core antigen. During the first 3 months of the program, 24,821 persons were tested for HBV and HCV infections. Among those tested, 1,084 (4.4%) had positive test results for HBsAg, and 1,075 (4.3%) had positive test results for HCV antibody (anti-HCV). Among those infected, 275 (25.4%) initiated treatment for HBV, and 163 (15.2%) initiated treatment for HCV, of whom 86.5% paid for medications and 13.5% received medications at no cost. Early results demonstrate willingness of patients to pay for treatment if costs are low, which can offset elimination costs. However, improvements across the continuum of care are needed to recover the upfront investment. Lessons learned from this program, including the effectiveness of using simplified test-and-treat guidelines, general practitioners in lieu of specialist physicians, and innovative financing to reduce costs, can guide similar initiatives in other countries and help curb the global epidemic of viral hepatitis, especially among low- and middle-income countries.

Viral hepatitis is a ubiquitous infectious disease. In 2015, an estimated 257 million persons had active HBV infection (*1*), 71 million persons had active HCV (*2*) infection, and approximately 1.3 million died from viral hepatitis and resulting liver disease (*1*). Despite the availability of hepatitis B vaccines and treatment for hepatitis B and C, few low- and middle-income countries have sustainable and scalable elimination programs. Lack of financing for large-scale testing and treatment is the main barrier to elimination, despite evidence of a positive return on investment (*3*). Certain countries require innovative approaches to financing because traditional funding mechanisms, such as donations and grants, remain largely unavailable for hepatitis elimination programs (*4*).

During 2016, Uzbekistan, with a population of approximately 30 million persons, had an estimated 2.5 million (8.3%) persons living with HBV infection (i.e., had a positive test result for HBsAg). Among those infected persons, 10% had a diagnosis, and only 0.5% (approximately 12,500) had been treated (*5*). An estimated 1.3 million (4.3%) persons were living with HCV infection (i.e., had a positive test result for HCV RNA), but only 5% of these persons had received a diagnosis, and only 2% of infected persons (approximately 26,000) had been treated (*6*). During 2017, the president of Uzbekistan issued a decree calling for the elimination of HBV and HCV infections to meet WHO’s 2030 hepatitis elimination targets (*1*). Initial assessments indicated that meeting these targets would cost US$1.3–1.7 billion (*7*) over 10 years, and the allocated funding of US$1.0 million per year for treatment and US$0.3 million per year for testing would be insufficient (*7*). To achieve elimination, an innovative catalytic funding model for providing low-cost, sustainable access to hepatitis B and C testing, care, and treatment was begun in 2019; this report describes program initiation and early findings.

The Center for Disease Analysis Foundation (CDAF)* developed a catalytic funding mechanism to allow low- and middle-income countries to fund HBV and HCV elimination programs with a low upfront investment and reduced overall cost. On December 6, 2019, CDAF began a pilot program to test the concept in Uzbekistan, in partnership with the Uzbekistan Research Institute of Virology and Ministry of Health. The catalytic funding model presumes that even among low- and middle-income countries, most people are willing to pay for HBV and HCV treatment if drug prices are below a catastrophic health care expenditure level^†^ (*8*). The model also presumes that a large portion of the population would be willing to take a free test. The catalytic investment is used to cover upfront costs for purchasing the first round of diagnostic tests and medications. All participants receive free testing. An estimated 20% of infected persons will receive free treatment, based on income level. A markup on treatment pricing for the 80% who can afford to pay for treatment funds the purchase of subsequent rounds of diagnostics and medication and repays the catalytic investment at the end of the program. Markups were calculated by dividing total project costs by the number of patients expected to pay for medicines (*7*). Performance indicators and minimum success criteria were developed to measure program performance relating to screening volumes, linkage and adherence to care rates, and repayment of the upfront capital investment (*7*).

The study protocol was approved by the national Institutional Review Board and the Uzbekistan Ministry of Health. Quality-assured medications and diagnostics were purchased at high volumes and low prices through the Global Procurement Fund, a nonprofit procurement service (*9*). The government waived most import duties and fees and provided human resources, clinic space, laboratory equipment, and disposables. A national pharmacy chain was contracted to sell medications at only a 5% markup.

Simplified testing algorithms were developed to minimize the number of tests required before starting treatment. Patients were tested using an HBsAg rapid diagnostic test (Alere Determine HBsAg 2, Alere Medical Company), followed by rapid human immunodeficiency virus (HIV) and creatinine tests (to assess renal function) if they had a positive HBsAg test. All patients with a positive HBsAg test, a negative HIV test, and normal renal function (estimated glomerular filtration rate [eGFR] >50 mL/min/1.73 m^2^) were determined to be eligible for treatment for hepatitis B infection. Patients who tested HBsAg positive were offered a 12-month prescription for tenofovir disoproxil fumarate, with instructions to return after 12 months for free follow-up tests (HBsAg, HIV, and creatinine). All patients who had a positive test result for HIV were referred to HIV clinics for treatment outside the Uzbekistan Hepatitis Elimination Program (UHEP).

An HCV rapid diagnostic test (InTec Products Inc.) was used concurrently with the HBsAg test to determine anti-HCV antibody positivity. Patients who had a positive anti-HCV antibody test had their blood drawn for HCV core antigen testing to confirm current infection (ARCHITECT HCV Antigen Assay, Abbott Laboratories), and for creatinine, aspartate aminotransferase (AST), and platelet tests. An AST to platelet ratio index (APRI) score was calculated to predict the likelihood of cirrhosis. Patients with APRI >1.5 or eGFR <30 mL/min/1.73 m^2^ (evidence of cirrhosis or impaired renal function) were referred to the Uzbekistan Research Institute of Virology for consultation with a specialist physician. All other patients with positive HCV core antigen test results were considered eligible for hepatitis C treatment and were offered a 3-month prescription for sofosbuvir/daclatasvir. All patients were instructed to return in 12 weeks, after completion of treatment, for a free HCV core antigen test to determine whether they had achieved a sustained virologic response (i.e., cure). Patients with cirrhosis and patients with impaired renal function were referred for treatment at the Uzbekistan Research Institute of Virology outside the UHEP.

In Tashkent, the capital city, 13 polyclinics were recruited to test an estimated 250,000 adults aged >18 years, over a 12-month period. Approximately 16,662 HBV patients and 6,866 HCV patients were estimated to be eligible for treatment during the program. Training on the use of rapid diagnostic tests, motivational interviewing (*10*), and patient registration was provided to all nurses participating in the program. Doctors were trained on the interpretation of laboratory results, medication contraindications, drug interactions, comorbidities, medication dosing, and patient registration. Handheld tablets or laptops and the open-source REDCap (Research Electronic Data Capture) patient registry were used to record patients’ consent, contact information, medical history, test results, and doctors’ notes.

Total UHEP costs, based on the calculated number of treated patients (Table), were estimated to be US$3,238,000, approximately one quarter of the estimated US$13,419,000 for a non-UHEP program of the same scope and size (*7*). The simplified testing and cost-containment measures (e.g., pooled procurement, waived duty taxes, and negotiated markups) substantially lowered total cost. Using the catalytic funding model, only US$1,616,000 in upfront costs were required to conduct the same program, with total program costs, including the upfront catalytic investment, covered by patient payments (*7*).

**TABLE:** Comparison of market pricing* for hepatitis B and hepatitis C tests, diagnostics, and treatments — Uzbekistan Hepatitis Elimination Program (UHEP), December 6, 2019–March 15, 2020

Item	Price (US$) per patient	
	Market pricing*	UHEP catalytic funding
Hepatitis B		
HBsAg testing	2.30^†^	Free^†^
Additional laboratory tests^¶^	55.25^§^	Free^¶^
Treatment (20% of patients)	365.00/yr**	Free**
Treatment (80% of patients)	365.00/yr**	180.00/yr**
Hepatitis C		
Anti-HCV testing	2.40^††^	Free^††^
Additional laboratory tests^§§^	43.50^¶¶^	Free^¶¶^
Treatment (20% of patients)	507.00***	Free***
Treatment (80% of patients)	507.00***	204.00***

**Abbreviations:** anti-HCV = hepatitis C virus antibody; HBsAg = hepatitis B surface antigen; HCV = hepatitis C virus.

* Market pricing reflects year 2019 costs.

^†^ Hepatitis B surface antigen rapid diagnostic test.

^§^ Hepatitis B virus enzyme linked immunosorbent assay, quantitative polymerase chain reaction, liver-staging, and blood workup.

^¶^ Human immunodeficiency virus rapid diagnostic test and creatinine.

** Tenofovir disoproxil fumarate.

^††^ HCV antibody rapid diagnostic test.

^§§^ Creatinine, aspartate aminotransferase, platelet, and hepatitis C virus core antigen to measure sustained virologic response.

^¶¶^ Viral load (times 2) and blood workup.

*** Sofosbuvir/daclatasvir 3-month prescription.

During December 6, 2019–March 15, 2020, a total of 24,821 persons were tested in Tashkent (approximately 10% of the number targeted for the year); 1,084 (4.4%) had positive test results for HBsAg and 1,075 (4.3%) had positive test results for anti-HCV (Figure). Fifty-one (4.7%) persons had positive test results for hepatitis B and hepatitis C. Three times more women (75.9%) than men (24.1%) participated in the program. A total of 428 (39%) persons who had positive test results for HBsAg and 291 (27%) who had positive test results for anti-HCV were already aware of their infection, and 176 (16.2%) patients who had positive test results for HBsAg and 128 (11.9%) who had positive test results for anti-HCV previously had been treated. Among persons who had a positive test result for anti-HCV, the hepatitis C core antigen positivity rate was 65.1% (68.4% if those already aware of their infection status were excluded). Overall prevalence of newly diagnosed HBV infection was 2.7%; prevalence was higher among men (4.7%; 271 of 5,798) than among women (2.1%; 391 of 18,595). Overall prevalence of newly diagnosed HCV infection was 3.2%; prevalence was higher among men (4.2%; 250 of 5,883) than among women (2.9%; 531 of 18,647).

**FIGURE:**
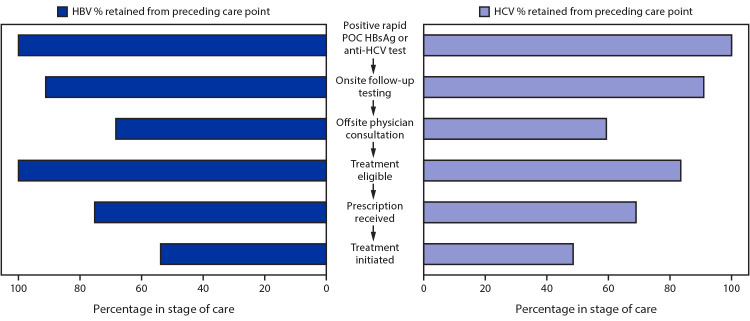
Percentage of persons who had positive test results for HBsAg or anti-HCV who were retained at each stage of care* — Uzbekistan Hepatitis Elimination Program — Tashkent, Uzbekistan, December 6, 2019–March 15, 2020 **Abbreviations:** anti-HCV = hepatitis C virus antibody; HBsAg = hepatitis B surface antigen; HBV = hepatitis B virus; HCV = hepatitis C virus; POC = point-of-care. * Treatment was initiated for 25.4% of all persons who had positive test results for HBsAg and 15.2% of all persons who had positive test results for anti-HCV.

Among the 1,084 patients who had positive test results for HBsAg, 988 (91.1%) received follow-up testing, as did 979 (91.1%) of the 1,075 patients who had positive test results for anti-HCV (Figure). Among those patients who received follow-up testing, 31.5% of those who had positive test results for HBsAg and 40.6% of those who had positive test results for anti-HCV did not attend their specialist physician consultation to discuss the test results and were lost to follow-up. Only 510 (75.3%) of the 677 treatment-eligible HBV patients and 335 (68.9%) of the 486 treatment-eligible HCV patients received prescriptions. Among all 1,084 patients who had positive test results for HBsAg and all 1,075 patients with HCV infection, 275 (25.4%) initiated treatment for HBV and 163 (15.2%) for HCV.

To succeed, the financial model needs a minimum of 55% of all patients who are diagnosed with chronic HCV and HBV to start and adhere to treatment (*7*). Initial data from the first 3 months of the programs show that only 23.0% of patients had started treatment, indicating that patient attrition in the cascade of care is currently too high to recover the upfront catalytic investment by program conclusion.

## Discussion

As of March 15, 2020, the UHEP in Tashkent had tested 24,821 persons for HBV or HCV. Among 2,159 persons with a positive test result, 438 had initiated treatment, including 275 (25.4%) persons who had positive test results for HBsAg and 163 (15.2%) persons who had positive test results for anti-HCV. Program success is attributed to free testing for all and lower treatment prices for those asked to pay, achieved through pooled procurement and negotiated markups. Although the overall male-to-female ratio in Tashkent is equal,^§^ women accounted for approximately three quarters of the tested population, but the prevalence of positive test results for HBV and for HCV was higher among men. The reasons for higher participation of women than men in the program are unknown.

Achieving WHO 2030 hepatitis B and C elimination targets will require substantial improvement in identifying and linking all eligible patients to treatment. This interim analysis identified a high rate of attrition in the cascade of care, with only 25.4% of persons with a positive HBsAg test result and 15.2% of persons with diagnosed HCV infection initiating treatment. The catalytic program funding model relies on 80% of infected persons paying for and initiating treatment; for the program to remain sustainable, treatment initiation must be increased. As the program moves forward, it will be important to identify reasons for attrition and to develop and implement strategies to improve rates of initiation and adherence to care.

The findings in this report are subject to at least two limitations. First, the population in Tashkent and the response to this program might not be representative of all of Uzbekistan. Second, the study was not designed to identify causes of nonparticipation, dropout, and loss to follow-up. Additional data are needed to help identify the barriers to recruitment and program participation.

Although some success was achieved during the first 3 months of the program, challenges to achieving the program targets remain. Despite notable reduction of costs and strong public participation in UHEP, improvements across the continuum of care are needed to fully recover program costs, repay the catalytic investment, and demonstrate a scalable and sustainable funding mechanism. CDAF is working with a consortium of international partners, including its technical advisory board, to address program challenges and introduce innovative strategies for success. Lessons learned from this program can guide similar initiatives in other countries, especially among low- and middle-income countries, to help curb the global epidemic of viral hepatitis in areas where donor support is limited. Catalytic funding models have the potential to substantially increase access to testing, diagnosis, and care and treatment for hepatitis B and hepatitis C.

**Summary**

**What is already known about this topic?**

Hepatitis B virus and hepatitis C virus infection prevalences are high in Uzbekistan, which lacks funding for meeting the World Health Organization’s 2030 elimination targets.

**What is added by this report?**

In December 2019, the Center for Disease Analysis Foundation initiated a pilot treatment program using innovative catalytic funding. During the first 3 months, >24,000 persons were tested and 438 initiated treatment, 87% of whom paid for medications and 14% received free medications.

**What are the implications for public health practice?**

Early results demonstrate willingness of patients to pay for treatment if costs are low, which can offset elimination costs. However, improvements across the continuum of care are needed to recover the upfront investment.

## Footnotes

* https://cdafound.org/.

† The catastrophic health-care expenditure level is a financial metric that determines the limit of out-of-pocket spending that will prevent a family from becoming financially destitute; the amount for Uzbekistan was calculated to be US$925, using per capita expenditure data from the World Bank and household size data from the United Nations.

§ https://www.indexmundi.com/uzbekistan/demographics_profile.html.
